# Effect of Maternal Diet on Maternal Milk and Breastfed Infant Gut Microbiomes: A Scoping Review

**DOI:** 10.3390/nu15061420

**Published:** 2023-03-15

**Authors:** Rachel Taylor, Deirdre Keane, Paulina Borrego, Kathleen Arcaro

**Affiliations:** 1 Department of Veterinary and Animal Sciences, University of Massachusetts, 240 Thatcher Road, Amherst, MA 01003, USA; 2 Science & Engineering Library, University of Massachusetts, Lederle Grad Research Ctr Low-Rise, 740 N Pleasant St Rm A273, Amherst, MA 01003, USA

**Keywords:** maternal diet, breastfeeding, breast milk microbiome, infant gut microbiome

## Abstract

While it is widely recognized that nutrition during pregnancy and lactation can affect the microbiome of breast milk as well as the formation of the infant gut microbiome, we are only just beginning to understand the extent to which maternal diet impacts these microbiomes. Given the importance of the microbiome for infant health, we conducted a comprehensive review of the published literature to explore the current scope of knowledge regarding associations between maternal diet and the breast milk and infant gut microbiomes. Papers included in this review assessed either diet during lactation or pregnancy, and the milk and/or infant gut microbiome. Sources included cohort studies, randomized clinical trials, one case-control study, and one crossover study. From an initial review of 808 abstracts, we identified 19 reports for a full analysis. Only two studies assessed the effects of maternal diet on both milk and infant microbiomes. Although the reviewed literature supports the importance of a varied, nutrient-dense maternal diet in the formation of the infant’s gut microbiome, several studies found factors other than maternal diet to have a greater impact on the infant microbiome.

## 1. Introduction

Human milk plays an important role in the development of the infant’s gut microbiome. Up to 88% of the genera are shared between human milk and the breastfed infant’s stool, supporting direct transmission [1]. In addition to direct transmission of the milk microbial community, breastfed infants receive nutrients, human milk oligosaccharides (HMOs), immune cells, antibodies, and secreted proteins that can further modulate the infant’s gut microbiome. The intestinal microbiome is critical for many aspects of infant health, including the development of the infant immune system, with lower risk for asthma, allergies, and autoimmune disease [2]. Given the relationship between human milk, the infant gut microbiome, and infant health, it is important to identify factors that alter milk and modify the infant microbiome. We are specifically interested in understanding the effects of maternal diet on the milk and infant gut microbiome as this information can be useful to both lactating parents and health professionals and may help advance infant feeding guidelines and provide postnatal intervention strategies [3].

While multiple factors, including lactation stage, mode of infant feeding (at the breast or pumped milk), gestational age at birth, and geographic location, influence the bacterial composition of human milk [3], maternal diet is of particular interest because it is perceived as a way that lactating parents can contribute to their infant’s health. Maternal diet may directly influence the diversity and relative abundance (RA) of bacterial taxa in the milk that the infant ingests, or alter other milk factors, such as HMOs, which support the development of the infant microbiome. In either case, the maternal diet has the potential to impact the infant’s intestinal health. Accordingly, there is concern that maternal consumption of some foods, such as artificially sweetened beverages (ASB), are negatively impacting the infant gut microbiome.

The scoping review is a useful approach for synthesizing evidence on a given subject and identifying possible knowledge gaps to inform future research. Despite great variation in methodology and study design within our topic of interest, this form of review allows us to summarize the main findings within the current body of literature. This review is specifically focused on studies directly assessing the relationship between maternal diet, either during gestation or postpartum, of lactating parents and the effects of their diet on either their milk or their infant gut microbiome. By using the inclusion criteria of either the milk or infant stool microbiome, this design allowed us to capture all studies investigating how maternal diet among lactating parents could alter the infant gut microbiome. In this review we sought to identify the study protocols and methods for both assessment of maternal diet and analysis of microbiomes.

## 2. Materials and Methods

### 2.1. Study Design

This scoping review was conducted using the Preferred Reporting Items for Systematic Reviews and Meta-Analyses extension for Scoping Reviews (PRISMA-ScR) checklist [4].

### 2.2. Identifying the Research Question

We sought to investigate the following question: “What is known about the influence of maternal diet on the breast milk microbiome and the breastfed infant gut microbiome?”.

The research question was refined, and the most appropriate databases identified to conduct and gather studies for review. Our research question involved linking three concepts: maternal diet, maternal milk microbiome, and breastfed infant gut microbiome.

### 2.3. Search Strategy and Eligibility Criteria

Two authors (D.K. and R.T.) conducted concurrent and comprehensive systematic electronic literature searches to find relevant studies. Three databases, Web of Science, PubMed, and Scopus, were searched on 29 September 2021. Two of the databases, Web of Science and PubMed, were accessed at the home institution, while access to Scopus took place at a neighboring consortium institution. An eduroam login account was used at the consortium institution to access the database due to closure of the physical library space to visitors.

The following search string was used to find articles with relevant terms in the article titles, abstracts, and keywords.

((“maternal diet” OR “maternal food” OR “maternal nutrition” OR “maternal dietary intake” OR “maternal nutritional status” OR “mother diet” OR “mother food” OR “mother nutrition” OR “mother dietary intake” OR “mother nutritional status”) AND (breastfeeding OR “breast milk” OR breastmilk OR “human milk” OR “pumped milk” OR “expressed milk” OR “milk composition” OR lactation OR “milk secretion”) AND (infant OR neonate OR neonatal OR newborn) AND (microbiome OR microbiota OR bacteria OR “gut microbiome” OR “microbiome clusters” OR microbial OR microbe OR gastrointestinal OR microflora)).

Specifically, the search parameters for each database were as follows: Web of Science: Topic search, PubMed: Title/Abstract search, and for Scopus: Title/Abstract/Keyword search. Because each database is formatted differently, these search parameters ensured consistency over database structures. There were no restrictions placed on the date of publication. Similarly, document types varied within databases, with PubMed offering the most granularity for targeted searching, see Table 1.

Individual database accounts were created by the authors prior to the search, and search results were saved to the account for backup. A shared Zotero account was created to deposit database results, and duplicate titles were removed.

An Excel spreadsheet was created listing the article publication year, author, and title for each reviewer. The spreadsheet was used to track articles meeting the criteria for inclusion. Specifically, inclusion criteria were journal articles published in English with no date restriction; review articles were excluded as well as books, letters, etc. Reviewers worked independently and averaged twenty-five articles reviewed per work session. Periodically, reviewers convened to discuss studies outside the original search language. For example, does body mass index (BMI) act as an indicator of maternal diet? The decision was made to exclude articles that only examined maternal use of supplements as part of dietary intake, as the researchers intended to focus specifically on food’s influence on the microbiome.

## 3. Results

### 3.1. Synthesis

**Figure 1:**
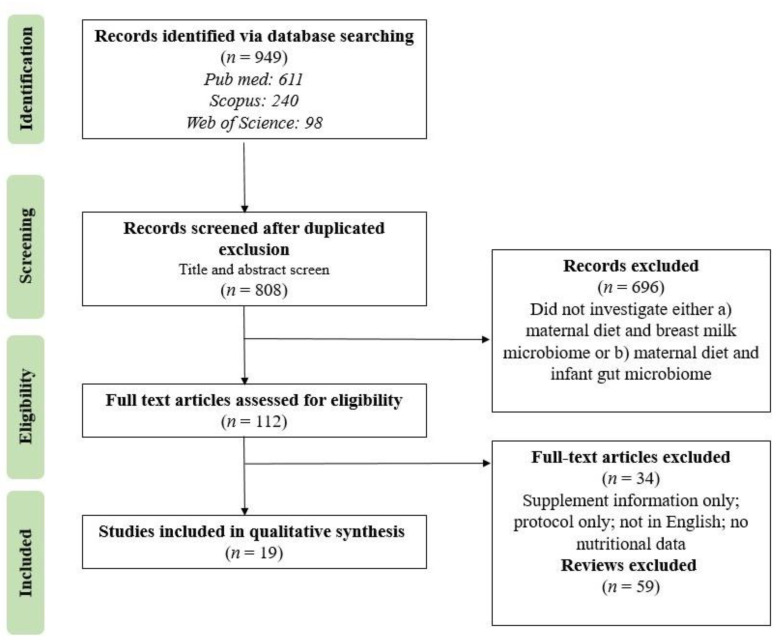
Flow diagram describing study selection process.

The database search identified a total of 949 records with our inclusion and exclusion criteria. After the removal of duplicates, 808 records remained for title and abstract screening. This resulted in the exclusion of 696 records that did not meet our criteria for investigation of maternal diet and/or the breast milk and infant gut microbiome. During screening, studies that did not investigate either (a) maternal diet and the breast milk microbiome or (b) maternal diet and the infant gut microbiome were excluded. Of the 112 papers subjected to full-text assessment, only nineteen publications met our eligibility criteria and were included in this scoping review. Exclusion criteria for eligibility included studies that provided only supplement information, only a protocol, were not in English, or did not provide nutritional data. The search results and exclusion details are outlined in the PRISMA flow chart (Figure 1).

A summary of the population and sample size, exposure variables of interest, study design, and relevant findings of the articles included is provided in Table 2. Fourteen of the papers were cohort studies [5,6,7,8,9,10,11,12,13,14,15,16,17,18], four were randomized controlled trials [19,20,21,22], and one was a case-control study [23]. The studies were conducted in Canada [10,12,14,23], the United States [6,11,18,20,21], Israel [5], the United Kingdom [16,22], China [15,17], Spain [7,8], Taiwan [9], Tanzania [19], and Brazil [13] and published between 2015 and 2021.

### 3.2. Analysis of Methodologies

#### 3.2.1. Sampling Procedures

Twelve studies collected milk samples [5,7,8,10,12,13,14,15,16,18,19,21], ten collected infant stool [5,6,8,9,11,14,17,20,22,23], and three collected both samples [5,8,14]. Additionally, three studies collected maternal stool [17,19,22]. One publication collected meconium from infants’ first diaper within 24–48 h of delivery [6] and one collected colostrum [15]. Four of the studies collected breast milk samples at multiple time points [5,18,19,21], and four collected infant stool at multiple time points [5,6,22,23]. Several studies requested or recommended morning sampling of breast milk [7,8,14,15]. One collected foremilk and hindmilk from feeds during a 24-h period [12]. Most did not specify the time of sampling. Among the papers reviewed, there were variations in sample collection and handling protocols as well as in sample storage.

#### 3.2.2. Microbiome Analysis

Of the 12 studies that collected milk, 11 examined the milk microbiome [5,7,8,10,12,13,15,16,18,19,21], and the remaining study examined HMOs [14]. Gut microbial analysis was assessed in all ten studies that collected infant stool and all three studies that collected maternal stool. Two studies examined both infant and maternal gut microbiomes [17,22].

The most frequent method of assessing the microbiome was sequencing of the 16S ribosomal RNA gene (16S rRNA). All but three studies conducted 16S rRNA sequencing. Of these three studies, Urwin et al. used fluorescence in situ hybridization with five genus-specific probes [22], Cortés-Martin et al. used quantitative polymerase chain reaction (qPCR) to investigate one specific microbial genus, *Gordonibacter* [8], and Seferovic et al. conducted both whole genome shotgun sequencing (WGS) and 16S rRNA gene sequencing. [21]. The 16S rRNA gene sequencing was conducted on samples for which the WGS resulted in less than 15,000 reads. The low reads with WGS (no amplification) are a consequence of the high abundance of human cells in milk, together with the relatively low level of bacteria.

The volume of milk used for extraction of DNA ranged between 300 mL and 10 mL, with seven studies using between 1 to 2 mL [7,8,10,12,13,15,16], three studies using 300 to 500 mL [5,19,21], and one study using between 0.5 and 10 mL [18]. Milk contains exfoliated breast epithelial cells and immune cells [24]; the density of these maternal cells in milk varies greatly among women and may dilute the ability to measure bacterial load, especially when small volumes of milk are assessed. No studies accounted for the host cells in milk. In contrast to milk, stool has a high bacterial load, and the starting volume may be less important as long as sufficient high-quality DNA is obtained. The volume of stool used for extraction of DNA was reported in only one study [22], with most studies referencing the manufacturer’s protocol.

#### 3.2.3. Nutrition Assessment

*Dietary intake assessment methods.* Nine studies used FFQs to assess maternal dietary intake [5,7,10,11,12,13,17,20,23]. Of these, five assessed gestational diet [7,11,13,17,23], one assessed postpartum diet [10], and three examined both [5,12,20]. Other methods used to gather dietary intake data were lifestyle questionnaires, 24-hr and 48-hr dietary recalls, 3-day dietary records, dietary screener questionnaire, and self-reported intake. Four papers conducted a diet intervention [8,19,21,22]. Dietary intake questionnaires differed in what food items or food groups were included.

*Anthropometric assessment methods.* Sixteen of the nineteen studies assessed maternal anthropometric characteristics, including BMI and gestational weight gain (GWG). These can be used as indicators of nutritional status in addition to direct assessment of dietary intake. Most assessed both maternal BMI and GWG [5,6,7,13,17,18,22,23]. Anthropometric measurement data were either self-reported or collected from clinical records. None of the papers in this review used more advanced anthropometric methods, such as bioelectrical impedance (BIA) or dual-energy X-ray absorptiometry (DEXA).

#### 3.2.4. Infant Feeding Assessment

Infant feeding factors, such as mode of breastfeeding and breastfeeding status, have been shown to influence both the breast milk and infant gut microbiomes. Of the papers reviewed, fourteen reported on infant feeding practices [6,7,8,9,10,11,12,15,20,21,22,23]. This assessment was conducted either by questionnaires, daily records, telephone interviews, or self-reporting. Three publications included only mothers who were exclusively breastfeeding [14,15,21]. Of the articles included in this review, LeMay-Nedjelski et al. [10] conducted the most rigorous assessment of infant feeding practices. Their questionnaire administered at three months postpartum asked about breastfeeding exclusivity, frequency of feeding at the breast versus bottle-feeding, as well as formula frequency and quantity.

## 4. Discussion

The aim of this scoping review was to identify known associations between (a) maternal diet and the breast milk microbiome and (b) maternal diet and the infant gut microbiome. Following the PRISMA guidelines, we identified nineteen articles that met the inclusion criteria. All nineteen reported maternal dietary intake, ten reported information on the milk microbiome, and eleven reported on the infant gut microbiome. Two papers reported information on both the milk and infant gut microbiomes.

### 4.1. What Impact Does Maternal Diet Have on the Milk Microbiome?

Of the ten studies that examined the effects of maternal diet on the milk microbiome, only one study reported no effect. In the RCT conducted by Bisanz et al., probiotic yogurt did not alter the milk microbiome but interestingly was associated with an increase in *Bifidobacterium* and a decrease in *Enterobacteriaceae* in the infant gut [19].

In contrast, the second study that assessed the effects of diet on both milk and infant microbiomes found diet altered only the milk microbiome. Babakobi et al. [5] studied the relationship between maternal diet during pregnancy and three months postpartum on the microbiomes of a cohort of 22 Israeli mother–infant dyads. They reported a negative correlation between and *Streptococcus* in milk maternal unsaturated fat and folic acid consumption and no significant associations between maternal diet and infant microbiome.

Two studies, Seferovic et al. [21] and Moossavi et al. [12] suggest mechanisms by which dietary factors may indirectly influence the breast milk microbiome composition by altering other milk components. Seferovic et al. conducted a crossover study that examined the impact of two dietary interventions on HMO composition and the milk microbiome in lactating women. The first diet intervention varied the carbohydrate source, glucose versus galactose (Glu/Gal), and the second varied the energy source, carbohydrates versus fat (Carb/Fat). The participants in the cohorts differed by lean and obese status and consisted of seven women each. The Glu/Gal intervention lasted 30–57 h and the Carb/Fat intervention was 8 days. Analysis of milk samples revealed a significant change in the HMO profile in the Glu/Gal cohort due primarily to an abundance of fucosidase producing bacteria. This study sheds light on how even short-term dietary changes can have a significant impact on the milk microenvironment. Moossavi et al. [12] analyzed the milk microbiome of samples collected from 393 women at 3–4 months postpartum. Their results show a significant association between maternal diet and BMI. Furthermore, a confirmatory factor analysis (CFA) revealed that maternal BMI is associated with changes in the overall milk environment that indirectly influence the milk microbiota. These studies show an indirect relationship between maternal diet, milk components such as HMOs and fatty acids, and the composition and diversity of the breast milk microbiome. For most studies, no intermediary changes in milk composition were examined or proposed.

Williams et al. [18] studied the associations between maternal nutrient intake and the milk microbiome in a cohort of 21 women between 2 and 6 months postpartum. They found the relative abundance of *Lactobacillus* to be negatively associated with maternal consumption of various micronutrients, such as thiamin, niacin, folate, vitamin B-6, and chromium. Additionally, the relative abundance of *Proteobacteria* was positively associated with a nutrient-rich diet and intake of various fatty acids. They also found that milk from mothers with male infants had higher *Streptococcus* and lower *Staphylococcus* than milk from mothers with female infants. The researchers assessed maternal dietary intake and collected milk samples at nine different time points, allowing for more robust analysis as compared to a single time point. This is important because 24-h dietary recalls are not always accurate representations of one’s diet, and long-term nutrient consumption may play a larger role in shaping the composition of the milk microbiome.

LeMay-Nedjelski et al. [10] also found a relationship between maternal dietary fat intake and specific genera in the milk, although different from the one Williams et al. [18] reported with *Proteobacteria*. LeMay et al. investigated the associations between maternal diet, infant feeding practices, and the milk microbial composition in a cohort of ninety-three women. The milk sampling and dietary assessment were conducted at the same time point of approximately 3 months postpartum. Their analyses revealed that maternal intake of fiber (especially from grains) and dietary fat were both associated with the milk microbiome composition at 3 months postpartum. Fiber intake from grains positively correlated with *Acinetobacter*, whereas total fiber was negatively correlated with *Streptococcus*. They found a positive association between *trans* fats and both *Staphylococcus* and *Gemella*. Higher monounsaturated fat intake was associated with an increased prevalence of both *Acinetobacter* and *Gemella*. Interestingly, polyunsaturated fat intake was negatively associated with *Acinetobacter*.

Padilha et al. [13] found, in their study of the milk microbiome of 94 Brazilian women at 1 month postpartum, that the intake of polyunsaturated/linoleic fatty acids during lactation showed a slight positive correlation with *Bifidobacterium* abundance in the milk. Intake of thiamin, riboflavin, and folate were negatively correlated with the *Enterococcus* genus. Vitamin C intake during pregnancy, rather than during the lactation period, was positively correlated with the *Staphylococcus* genus. In the cluster of women with *Staphylococcus* as the predominant bacterial genera, the authors also observed higher intake of pectin and lycopene. These nutrients, including Vitamin C, are all common to citrus fruits. This dietary pattern during pregnancy and the associated milk microbiome effects suggests beneficial development for the infant’s immune system.

Cortes-Macías et al. [7] studied the breast milk microbiome in relation to maternal diet in 120 women from the MAMI cohort study [25], with milk samples collected between 7 and 15 days after birth. Maternal diet was grouped into two clusters, the first characterized by high intake of plant protein, fiber, and carbohydrates and the second by high intake of animal protein and lipids. The genera *Lactobacillus*, *Bacteriodes*, and *Sediminibacterium* were found in significantly lower abundances among the group of women in Cluster II, who gave birth by C-section and had antibiotic exposure. This finding is an example of how diet and other maternal factors interact to influence the milk microbiome composition.

In their 2020 study, Shenker et al. [16] conducted 16S rRNA sequencing on milk samples from 46 of the 62 participants who provided milk. Maternal intake of alcohol and soy were correlated with 11 and 12 taxonomic features. Intake of folic acid correlated with one genus, *Anaerococcus*. The authors note that one individual self-reported a much higher alcohol intake than the rest of the cohort, so this outlier may be responsible for identification of false positive correlations. Therefore, they suggest that the relationship between alcohol consumption and the milk microbiome be explored further in a larger cohort.

### 4.2. What Impact Does Maternal Diet Have on the Breastfed Infant Gut Microbiome?

The second aim of this review was to identify associations between maternal diet, both during pregnancy and while breastfeeding, with the development of the infant gut microbiome. Eleven studies examined this relationship, and eight identified maternal dietary factors associated with concentrations of specific taxa in the infant gut. For example, Urwin et al. [22] found a lower abundance of the *Atopobium* bacterial cluster in the stool of infants whose mothers consumed salmon on a weekly basis during pregnancy. This finding was consistent among samples collected at 7, 14, 28, and 84 days postpartum, “especially those who were not exclusively breastfed”, indicating that the mode of infant feeding plays a role in the impact of salmon consumption during pregnancy on the infant gut microbial composition.

Lundgren et al. [11] also reported an association between the maternal consumption of seafood and the infant gut microbiome. While assessing the effects of maternal diet on the infant gut microbiome in 6-week-old infants from the New Hampshire Birth Cohort Study, Lundgren et al. found that maternal seafood consumption was positively associated with the genus *Streptococcus* in the infant gut and negatively associated with the species *Clostridium neonatale* in the gut of infants born by Cesarean section. A decrease in *Clostridium neonatale* may be beneficial for infants as that species is frequently found in patients with necrotizing enterocolitis. The authors noted that some effects of maternal diet were more apparent in infants that were exclusively breastfed; however, they did not draw conclusions based on the mode of infant feeding as most infants were fed a combination of breast milk and infant formula. Instead, they observed that mode of delivery alters the way in which maternal diet affects the establishment of the infant gut microbiome. For instance, Lundgren et al. unexpectedly found a negative association between maternal diets high in fruits and vegetables and the beneficial microbe *Bifidobacterium* in vaginally born infants and a positive association between maternal diets high in red and processed meats and *Bifidobacterium* in infants born via Cesarean section. These results highlight that while maternal diet is associated with the infant gut microbiome, additional factors, such as mode of delivery, also contribute to the development of the infant gut microbiome.

Bisanz et al. [19] assessed the influence of maternal consumption of probiotic yogurt with *Lactobacillus rhamnosus* GR-1 supplemented with Moringa plant on the breastfed infant gut microbiome. They found that the maternal consumption of yogurt was associated with an increase in the relative abundance of *Bifidobacterium* and a decrease in the relative abundance of *Enterobacteriaceae*. The study also assessed the microbiota at multiple maternal body sites but observed no significant effects on the breast milk microbiome.

In their 2021 pilot study, Fan et al. [9] examined the effect of maternal fruit and vegetable consumption on the infant gut microbiome at 2 months postpartum. They found an increased abundance of the beneficial genera *Cutibacterium*, *Parabacteroides*, *and Lactococcus* in the gut of infants whose mothers had high gestational consumption of fruits and vegetables (n = 13) as compared to those with low gestational consumption of fruits and vegetables (n = 26).

Wang et al. [17] examined the relationship of maternal diet and alcohol consumption during pregnancy with the infant gut microbiome in 29 Chinese mother–infant dyads. While the authors found no effect of maternal diet on the infant microbiome, maternal alcohol consumption (categorized as either never: n = 19 or rarely, some of the time, and most of the time: n = 10) was associated with increased microbial diversity in infant stool. However, the small sample size and the categories of alcohol consumption warrant caution in generalizing these results.

Other studies investigated the effect of maternal artificial sweetener and fat intake on the infant gut microbiome. For example, Laforest-Lapointe et al. [23] found that maternal consumption of artificially sweetened beverages (ASB) during pregnancy impacted the infant gut microbiome. Maternal ASB intake resulted in a depletion of *Bacteroides* and an increase in *Prevotella copri* in the infant gut. Interestingly, they also reported a positive association between maternal ASB consumption during pregnancy and infant BMI at one year.

Chu et al. [6] examined the effects of maternal high-fat diet during gestation on the infant gut microbiome from birth to 6 weeks of age. Of the 163 participants enrolled in the cohort, 26 participants reported dietary intake of fat that differed significantly from the mean. Those 26 participants were divided into a control group (n = 13) and a high-fat group (n = 13). Other confounding factors, such as sugar and fiber intake, pre-pregnancy BMI, and mode of delivery, did not significantly differ between the two groups. Meconium samples were collected at the time of delivery, and infant stool samples were collected 4–6 weeks later in order to analyze the infant gut microbiome. The authors found that a gestational maternal high-fat diet was significantly associated with an increase in *Enterococcus* and a relative depletion of *Bacteriodes* in the infant meconium. Analysis of the infant stool collected at 6 weeks showed the correlation between *Bacteriodes* and maternal gestational fat intake persisted, while the *Enterococcus* correlation was no longer significant. The depletion of *Bacteriodes* may affect infant energy as the species is important for metabolizing complex polysaccharides. These two studies [6,23] provide evidence that the beneficial infant gut genus *Bacteriodes* is significantly depleted as a result of both maternal ASB consumption and high-fat diet.

Quin et al. [14] investigated the influence of diet-derived HMOs on the infant gut microbiome. They found maternal fruit and vegetable consumption to be associated with increased abundance of several HMOs. In addition, they showed, for the first time, that diet-derived Neu5gc (found in red meats), is incorporated into HMOs, and they observed a positive association between Neu5Gc and *Bacteroides* spp. abundance in infant stool. However, they acknowledge that *Bacteriodes* spp. metabolize a variety of HMOs, and therefore the increase in abundance may not be due to the diet-induced HMOs.

Finally, three studies reported no significant effect of maternal diet on the breastfed infant’s gut microbiome. Babakobi et al. [5] assessed the microbiome of breast milk and infant stool in 22 dyads at three time points. Maternal diet was assessed with a long-term eating pattern FFQ. While they found that maternal diet altered the milk microbiome, they observed few similarities between the milk and infant gut microbiome and concluded that there was no association between maternal diet and the composition of the infant gut microbiome. Savage et al. [20] also found no association between maternal diet and the infant gut microbiome but had a much larger sample size of 323 participants. Of the 323 infants in the study, ninety-five were exclusively breast fed and 169 were exclusively formula fed. Savage et al. found that the differences in feeding methods, rather than in maternal diet, had strong associations with changes in the infant gut microbiome.

Cortés-Martín et al. [8] explored the role of maternal walnut intake on the urolithin-producing bacterium, *Gordonibacter,* in the infant gut. Breastfeeding mothers ate walnuts (30 g per day) for 3 days, and then urolithins (produced by maternal gut *Gordonibacter*) were measured in their milk. Interestingly, neither vaginal delivery nor breastfeeding were required to establish *Gordonibacter* in the infant, yet the type of urolithins present in the milk were associated with the timing of establishment of infant *Gordonibacter.* This appears to be a case in which maternal diet alters the maternal gut microbiome, and through proximity, the mother transmits the *Gordonibacter* to the infant.

## 5. Conclusions

The objective of this scoping review was to examine the existing literature assessing the effects of maternal diet on the milk and infant gut microbiomes. Compliance with the PRISMA-ScR guidelines was followed throughout the entire review process. Surprisingly, we identified only nineteen studies that met our review requirements, indicating that additional research is needed. Sample sizes ranged from 14 to 393, with eleven studies having less than 100 participants. Half of the studies examined only the milk or only the infant gut microbiome, and only two studies assessed both microbiomes. Overall, the results from the reviewed studies support two conclusions: (1) Maternal diet can impact the milk microbiome, and (2) Maternal diet can impact the infant gut microbiome, both negatively and positively. The extent to which maternal diet influences the infant microbiome through microbial changes in milk remains to be determined.

This review has several limitations. First, we defined maternal diet as “food” and did not include supplements. Second, the review was restricted to papers originally published in English. Third, only publications prior to 29 September 2021, were examined, as substantial time was required for full text assessment of 112 publications.

Future research on this topic should be designed to collect and analyze both maternal milk and infant stool samples and include larger sample sizes. Dietary assessments should include supplements. Given the positive results of cohort studies, clinical trials to understand how maternal diet can improve the infant gut microbiome are warranted.

## Figures and Tables

**Table 1:** Databases and document types included in the search.

Database	Document Type Included
Web of Science	Abstract, Article, Case report, Correction, Early Access, Retraction, Review
Scopus	Article, Review, Short survey, Erratum
PubMed	Case Reports, Classical Article, Clinical Study, Clinical Trial Protocol, Clinical Trial, Phase I, II, III, IV, Controlled Clinical Trial, Corrected and Republished article, Journal Article, Letter, Multicenter Study, Preprint, Published Erratum Research support, Research Support, NIH, Extramural Research Support, NIH, Intramural Research Support, Non-US Gov’t Research Support, US Gov’t, Non-PHS Research Support, US Gov’t, PHS Research Support, US Gov’t Retracted Publication Retraction of Publication, Technical Report, Twin study, Validation Study

**Table 2:** Studies assessing the relationship between maternal diet and maternal or infant microbiome.

Reference	Exposure Variables	Study Design	Population & Sample Number	Maternal Nutritional Assessment	Milk	Stool Samples	Microbiome Analysis	Main Relevant Finding
Babakobi et al., 2020 [5]	Maternal nutrition during pregnancy and 3 months postpartum	cohort study	n = 22 mother– infant dyads	food frequency questionnaire (FFQ)	1 week 1 month 3 months postpartum (pp)	Infant: 1 week 1 month 3 months	Power Soil DNA Kit (MoBio) 16S rRNA regions V3 and V4 (341F/806R) MiSeq (Illumina) PEAR database	Negative correlation between maternal unsaturated fat consumption and *Streptococcus* in milk at one month. No significant associations between maternal diet and infant gut microbiome.
Bisanz et al., 2015 [19]	Probiotic yogurt: *Lactobacillus rhamnosus*, plus ground *Moringa olifera*	randomized clinical trial (RCT)	n = 56 mother– infant dyads	Dietary recalls	3 days pp and 1 week to 1 month after first sample	Infant & Maternal: 3 days pp and 1 week to 1 month after first sample	Power Soil DNA Kit (Mobio) 16S rRNA V4 (515F/806R) MiSeq (Illumina) Greengenes	Maternal yogurt consumption did not alter milk microbiome but was associated with changes in the infant gut microbiome: increase in *Bifidobacterium* and decrease in *Enterobacteriaceae.*
Chu et al., 2016 [6]	Maternal high-fat diet during gestation and lactation	cohort study	n = 163 mother– infant dyads	rapid dietary questionnaire	No	Infant: meconium and stool at 4–6 weeks of age	Power Soil DNA Kit (MoBio) 16S rRNA, V3-V5 Pyrosequencing with 454-FLX Titanium QIIME	Maternal consumption of a high-fat diet during gestation was associated with a depletion of *Bacteriodes* in meconium that persisted at 6 weeks.
Cortes-Macías et al., 2021 [7]	Maternal diet, mode of delivery, and antibiotic exposure	cohort study	n = 120 lactating women	FFQ	7–15 days pp	No	MasterPure DNA Extraction Kit (Epicentre) 16S rRNA regions V3-V4 SILVA database	Identified significant associations between dietary nutrients and specific breast milk microbial genera (2 clusters).
Cortés-Martín et al., 2020 [8]	Walnut intake (30g daily for 3 days)	cohort study	pilot study: n = 11 full study: n = 30 mother– infant dyads	30 g walnuts/day dietary intervention	pilot: 2 weeks to 24 months pp full study: within 1 year pp	Infant: within 1 year of birth	Milk:MasterPure Complete DNA & RNA Purification Kit; Infant stool: Nucleospin tissue DNA purification kit 16S rRNA qPCR for *Gordonibacter*; ABI 7500 real-time PCR System	Colonization of the infant gut with *Gordonibacter* occurred during the first year and was not dependent on breastfeeding but was influenced by maternal urolithin metabotype
Fan et al., 2021 [9]	High or low fruit and vegetable gestational intake	cohort study	n = 39 mother– infant dyads	3-day dietary record	No	Infant: 2 months of age	Qiagen DNA Mini kit 16S rRNA V3–V4 (341F/805R) MiSeq 2000 SILVA database	Infant gut microbiome clustered differently for high & low maternal fruit and vegetable consumption. Higher maternal intake of fructose, dietary fiber, folic acid, and ascorbic acid negatively associated with unhealthy infant gut microbiome.
Laforest-Lapointe et al., 2021 [23]	Maternal consumption of artificially sweetened beverages (ASB)	case-control study	n = 100 mother– infant dyads	reported ASB consumption	No	Infant: 3 and 12 months of age	DNeasy Power Soil Kit (Qiagen) 16S rRNA V4 (F515/R806) MiSeq	Maternal ASB consumption was associated with higher infant BMI, and infant BMI was associated with the microbiome composition at 12 months, but not at 3 months of age. Estimated impact of ASB consumption on the infant microbiome was notably smaller than other known drivers (breastfeeding, birth mode, ethnicity, infant age, and antibiotics).
LeMay-Nedjelski et al., 2021 [10]	Maternal diet and infant feeding practices	cohort study	n = 93 lactating women	FFQ	3 months postpartum	No	NucleoSpin Fod DNA Isolation kit (macherey-Nagel) 16S rRNA V4 (515F/805R)	Maternal intake of polyunsaturated fat and fiber was associated with increased alpha diversity in milk microbiota at 3 months pp. Infant feeding practices were associated with milk microbiome.
Lundgren et al., 2018 [11]	Maternal diet during pregnancy	cohort study	n = 145 mother– infant dyads	FFQ	No	Infant: 6 weeks of age	Zymo DNA Extraction kit 16S rRNA V4–V5	Identified three clusters of the infant gut microbiome in vaginally delivered infants and caesarean delivered infants. Cluster 2 (high abundance of *Streptococcus* and *Clostridium*) was associated with maternal fruit consumption.
Moossavi et al., 2019 [12]	Maternal and infant early-life factors	cohort study	n = 393 mother– infant dyads	FFQ	3–4 months pp	No	Quick-DNA Fungal/Bacterial extraction kit 16S rRNA V4 (515F/806R) MiSeq	Factor analysis showed that maternal diet influences BMI, which indirectly affects milk microbiota by altering factors in milk. Mode of breastfeeding was associated with breast milk microbiota composition. Maternal BMI and parity associated with milk microbiota in a sex-specific manner.
Padilha et al., 2019 [13]	Maternal diet during pregnancy and first month of lactation	cohort study	n = 94 lactating women	quantitative FFQ	~30 days pp	No	QIAmp DNA Mini kit 1Nested PCR 16s rDNA Nested PCR 1st (341F/806R) 2nd (515F/806R) MiSeq	Vitamin C intake during pregnancy was correlated with the presence of *Staphylococcus* genus. Intake of PUFAs and linoleic acid during lactation were correlated with *Bifidobacterium*.
Quin et al., 2020 [14]	Maternal diet & diet-induced HMO alterations	prospective cohort study	n = 109 mother– infant dyads	24-h dietary recalls	5 months pp	Infant: 5 months of age	QIAMP DNA Stool Mini kit 16S rRNA V3–V4 (341F/805R) MiSeq	Maternal diet affected HMOs in breast milk. HMOs influenced the infant microbiome composition. Maternal secretory status also had a modest effect on infant microbiome.
Sakwinska et al., 2016 [15]	Lactation stage & aseptic vs. non-aseptic collection	cohort study	n = 90 lactating women	lifestyle questionnaire	0–4 days 5–11 days 2 months postpartum	No	DNA Stool Mini kit (Qiagen) Or Fast DNA SPIN kit for soil (MoBio) 16S rRNA V4	Confirmed the presence of the dominant species in breast milk such as *Streptococci* and *Staphylococci* & low abundance of *Bifidobacteria* and *Lactobacilli*. Results suggest that the microbiota of milk from Chinese lactating mothers is similar to that observed from other geographic locations.
Savage et al., 2018 [20]	Diet during pregnancy & infancy (breastfed versus formula)	RCT	n = 323 mother– infant dyads	FFQ	No	Infant: 3–6 months of age	16S rRNA	No significant association between maternal diet during pregnancy and infant microbiome. Infant’s diet impacted infant gut microbiome.
Seferovic et al., 2020 [21]	Maternal carbohydrate (glucose/ galactose) & energy sources (high carb/high fat)	crossover study	n = 14 lactating women; 7 per intervention	Two crossover dietary interventions: Glu/Gal & Carb/Fat	8–11 weeks pp	No	WGS sequencing	Short-term diet of 30–57 h altered HMOs, which in turn altered the microbial gene expression.
Shenker et al., 2020 [16]	Self-reported lifestyle and dietary factors	cohort study	n = 62 mother– infant dyads	self-reported intake	3–48 months pp	No	16S rRNA	Maternal intake of alcohol and soy were correlated with 11 and 12 taxonomic features, respectively. Intake of folic acid correlated *Anaerococcus.* Results suggest stability in human milk composition for up to 24 months.
Urwin et al., 2013 [22]	Maternal consumption of 150 g of salmon twice per week during pregnancy	RCT	n = 123 mother– infant dyads	diet intervention, two 150 g portions of salmon/week from week 20 gestation to delivery	No	Infant: 7, 14, 28, and 84 days of age Maternal: 38 weeks gestation	Five 16S rRNA probes to genus-specific microbes Fluorescence in situ hybridization	Increased salmon consumption during pregnancy had no significant effects on maternal microbiome. Salmon consumption during pregnancy was associated with lower abundance of the *Atopobium* cluster in stool of infants in the first 84 days postpartum, especially those who were not exclusively breastfed.
Wang et al., 2021 [17]	Maternal diet and alcohol consumption during pregnancy	cohort study	n = 29 mother– infant dyads	alcohol consumption questionnaire	No	Infant: within 48 h after birth; Maternal: late pregnancy	E.Z.N.A. soil DNA kit (Omega Bio-tek) 16S rRNA V3-V4 (338F/806R) MiSeq SILVA	Gut alpha diversity differed between alcohol & no alcohol groups. Pregnant mothers who consumed alcohol had higher diversity, and similar results were shown in newborns. Maternal dietary intake of meat, eggs, and soybean products during pregnancy were associated with the infant gut microbiota.
Williams et al., 2017 [18]	Maternal nutrient intake, time postpartum, delivery mode, and BMI (kg/m^2^)	cohort study	n = 21 lactating women	24-h dietary recalls	2 to 6 months pp	No	Enzymatic lysis & physical disruption, QIAmp DNA Mini Kit (Qiagen) 16S rRNA V1–V3 (F27/R534) MiSeq Ribosomal Database Project	Maternal consumption of thiamin, niacin, folate, and vit. B-6, and chromium was negatively associated with *Lactobacillus* in milk. Positive association between a nutrient-rich diet and maternal intake of various fatty acids with *Proteobacteria* in milk.

## Data Availability

No new data were created or analyzed in this study. Data sharing is not applicable to this article.
